# The allelochemical tannic acid affects the locomotion and feeding behaviour of the pond snail, *Lymnaea stagnalis,* by inhibiting peripheral pathways

**DOI:** 10.1007/s10158-019-0229-7

**Published:** 2019-08-22

**Authors:** Ágnes Vehovszky, Réka Horváth, Anna Farkas, János Győri, Károly Elekes

**Affiliations:** MTA Centre for Ecological Research, Balaton Limnological Institute, Tihany, 8237 Hungary

**Keywords:** Allelochemicals, Tannic acid, *Lymnaea*, Feeding, Locomotion, Peripheral pathways

## Abstract

(1) The effect of tannic acid (TA), a dominant component of plant allelochemicals, was investigated on the locomotion and feeding of the pond snail, *Lymnaea stagnalis*. The effect of TA on the neuronal background underlying feeding activity was also analysed. (2) TA affected the spontaneous locomotion and of juvenile snails in a concentration-dependent way. Low (10 μM) TA concentration resulted in an increased (sliding or swimming) activity compared to the control; meanwhile, high (100 μM) TA concentration inhibited the locomotion of the animals. (3) Low (10 μM) TA concentration increased the frequency of sucrose-evoked feeding of intact animals, whereas high (100 μM) TA concentration resulted in significantly longer feeding latency and decreased feeding rate. The feeding changes proved to be partially irreversible, since after 48 h maintained in clear water, the animals tested in 100 μM TA previously still showed lower feeding rate in sucrose. (4) Electrophysiological experiments on semi-intact preparations showed that application of 100 μM TA to the lip area inhibited the fictive feeding pattern of central neurons, the cellular response to sucrose. (5) On isolated CNS preparation, 100 μM TA applied in the bathing solution, however, failed to inhibit the activation of the central feeding (CPG) interneurons following application of extracellular dopamine. Our results suggest that TA affects both afferent and efferent peripheral functions in *Lymnaea*. TA reduces feeding activity by primarily blocking feeding sensory pathways, and its negative effect on locomotion may imply sensory pathways and/or ciliary activity.

## Introduction

### Tannins

Tannins, a heterogenous group of high molecular weight (500–3000 Da) water-soluble compounds, members of the even more complex family of polyphenolics, are among the most studied plant secondary metabolites from a chemical–ecological aspect (Salminen and Karonen 2011). The synthesis of these phenolic compounds is not restricted to plant species found in particular habitats, as it is also present in cereals, legumes, nuts, fruits, vegetables, beverages and red wines (Scalbert and Williamson 2000; Serrano et al. 2009), confirming their abundance in our diet (approx. 0.1–1 g/day). Tannins play an essential role in the survival of plants because of their protective capacity against various biotic and abiotic stressors (Haukioja 2005; Smeriglio et al. 2017). Their biological activity is based on their capability of precipitating macromolecules (particularly proteins) and metals, and to cause oxidative stress by auto-oxidation or enzymatic processes (Salminen and Karonen 2011). Polyphenolic compounds including tannins have numerous different effects depending on both their chemical structure and environment. For example, they may evoke contradictory effects in the mostly alkalic gut of insects and the acidic alimentary tract of mammals (Salminen and Karonen 2011). This may partially explain why tannins have anti-nutritive effects on numerous herbivorous insects and antimicrobial activities on bacteria (Appel and Schultz 1994; Graca and Barlocher 1999; Marin et al. 2015), but exert positive dietary effect in mammals, including human (Scalbert and Williamson 2000; Harbertson et al. 2008; Serrano et al. 2009; Sieniawska 2015). Intracellularly, tannins are also characterized by a high diversity of negative effects (Anku et al. 2017), as well as positive pharmacological and biochemical actions, including antioxidant and radical scavenging and anti-cancerous effects (Gollucke et al. 2013; Sieniawska 2015; Skrovankova et al. 2015; Smeriglio et al. 2017).

In natural waters, the presence of tannins together with an even more complex group of humic substances is resulted by vegetative degradation (decomposition of leaves, roots, etc.). Their concentrations vary in different aquatic environments with the highest level detected in the coloured water of bogs, while in some Canadian rivers and lakes tannin concentration was measured between 0.5 and 4 mg/l (Afghan and Chau 2017). We should also note that at present no generally established analytical methods are available to detect tannins alone (Salminen and Karonen 2011). The living tissues of several aquatic plants (incl. *Myriophyllum*) were shown to contain high amount of polyphenolics (3–150 mg/g dry weight), and also release them in the environment (2–4 mg/g dry wt of tannic acid equivalent) in a 10-day period (Gross et al. 1996; Choi et al. 2002; Li et al. 2004).

In addition to their deterrent functions (Coley et al. 1985), hydrolysable and condensed tannins can perturb the development and growth of numerous aquatic herbivorous insects (Feeny 1976; Choi et al. 2002). Tannins can reduce the size of the female pupae of the autumnal moth, *Epirrita autumnata*, resulting in a decreased number of laid eggs and deterioration of the physical state of the adult insect (Haukioja and Neuvonen 1985; Tikkanen et al. 2000), and also causing a higher risk of predation mortality (Feeny 1976).

Both terrestrial and aquatic molluscs provide good models to indicate toxicity and for the analysis of toxic effects on their nervous systems (Salánki et al. 2003; Rittschof and McClellan-Green 2005; Das and Khangarot 2011). Although different allelochemicals, among them tannins, are suggested to be a molluscicide effect in case of snails (Singh et al. 1996; Rawani et al. 2014), our knowledge is limited regarding the possible mode of action of their harmful/sublethal effects on behaviour and neuronal mechanisms.

In our present study, two forms of behaviour (locomotion and feeding) were monitored in the presence of the commercially available tannic acid (TA). Both locomotion and feeding, as well as the neuronal basis of their regulation, have widely been studied in gastropods, including our model animals, *Lymnaea stagnalis* (for review see Chase 2002; Benjamin 2012).

### Locomotion

In gastropods two major forms of locomotion, crawling and swimming, have been distinguished (for review see Chase 2002). Crawling, mentioned also sometimes as sliding, is typical for both terrestrial and aquatic gastropod species, although the cellular execution of the movement is different; in case of terrestrial species it is based on the rhythmic contraction of the foot musculature (*Helix, Limax*), whereas in the case of aquatic animals the forward movement is performed by the beating of cilia covering the sole of the foot (*Lymnaea, Planorbis*). Swimming is mainly confined to Opistobranchiata, performing the movement either by paddles or wings (*Clione*), whereas some benthic species have a sliding movement, moving by a typical undulation of the whole body resembling crawling during escape (*Tritonia*). In the case of *Aplysia* species, some like *A. californica* perform crawling by using their foot, whereas others like *A. fasciata* use a pair of parapodia originating from the foot for swimming.

The target of our present study, the pond snail, *Lymnaea stagnalis*, as an aquatic species displays mainly ciliary-based crawling (sliding) (Chase 2002). In addition, we could distinguish three further forms of locomotion during our present research: swimming, floating and sticking. These forms of locomotion and their regulatory mechanisms have not yet been studied in *Lymnaea*.

The neuronal regulation of locomotion is based on motoneurons located in the pedal ganglia controlled by a central pattern generator (CPG) network in the CNS. Regarding the transmitters involved, two neuronal mechanisms have been identified. The ciliary movements are modulated by serotonergic mechanisms originating from the central (pedal) neurons (Sidorov and Kazakevich 2001; Pavlova 2010), while the foot muscle contractions are under dopaminergic regulation (Pavlova 2013, 2019). The spontaneous locomotion of *Lymnaea* can easily be monitored and quantified (Pyatt et al. 2002; Dhara et al. 2017; Ford et al. 2018).

### Feeding

The feeding behaviour of *Lymnaea* is a good indicator when detecting neuronal changes behind neurotoxic effects (Kemenes et al. 1990; Vehovszky et al. 2007), ageing (Arundell et al. 2006) or learning processes (Staras et al. 1998).

Consummatory feeding of *Lymnaea* is a result of the highly coordinated contractions of the buccal musculature, executed in a sequence starting by radula protraction, followed by radula retraction and then swallowing. This rhythmic activity of feeding is regulated by a central pattern generator (CPG) network of interneurons located in the CNS (Benjamin 1983; Benjamin and Elliott 1989). The neurons of the CPG activate each other as well as other members (both interneurons and motoneurons) of the feeding system by a cyclic pattern of synaptic inputs. While recording intracellularly, a rhythmic activity pattern called fictive feeding allows to easily identify the central feeding neurons and analyse their possible modulation in both semi-intact and isolated CNS preparations. Activating the feeding rhythm of the central network, moreover, does not necessarily requires peripheral inputs, as fictive feeding can also be triggered in the isolated CNS preparation either by intracellular stimulation of higher-order feeding interneurons (Elliott and Benjamin 1985; McCrohan and Kyriakides 1989; Vehovszky and Elliott 2001) or by extracellularly applied monoamines (dopamine [DA], octopamine, Kyriakides and McCrohan 1989; Vehovszky et al. 1998).

### Aims

Based on the above, tannic acid (TA), a specific plant polyphenol (belonging to the galloylglucose family), was selected for our present study to clarify its potential effect on different behaviours (locomotion and feeding) of a model invertebrate, the pond snail, *Lymnaea stagnalis* L. Applying electrophysiological methods, we also analysed the TA-evoked changes in the neuronal network responsible for the regulation of feeding activity.

## Materials and methods

### Animals

Adult snails were collected locally in the Lake Balaton region (Hungary) between May and September 2018, maintained in 120-L glass aquaria filled with aerated filtered Balaton water and fed on lettuce ad libitum. The Balaton water taken from the open water area (about 100 m from the shore, depth 3–3.5 m) was free from macrophytes, the natural sources of polyphenols in freshwater. Juvenile specimens of *Lymnaea stagnalis* originated from our laboratory-bred populations. All the experimental animals were isolated 2–4 days before testing and placed in 15-L containers filled also with aerated filtered Balaton water, in the absence of food.

### Chemicals

All chemicals used for the preparation of the *Lymnaea* physiological saline (Magoski et al. 1995), as well as tannic acid (TA), dopamine hydrochloride (DA) and sucrose were purchased from SIGMA (Budapest, Hungary). The testing solutions containing 10 or 100 μM TA were freshly prepared in Balaton water immediately before the locomotion and feeding assays. Sucrose solution (100 mM) was prepared in Balaton water and used during the feeding tests, and for experiments on semi-intact preparations it was applied on the lip area. For the electrophysiological test on isolated CNS preparations, DA was applied from a pipette directly into the experimental chamber, while TA prepared in *Lymnaea* physiological saline was applied in the bath by perfusion.

### Behavioural tests

#### Locomotion

In the locomotion study, P2 juvenile snails (*Lymnaea stagnalis*) were used (for staging see Croll and Chiasson 1989). The reason for using juveniles and not adults was that observing and tracing the different (active and passive) forms of locomotion, which are already both identical with those of the adults at this time of development, were easier compared to observing the larger size adult animals. As already mentioned above (see Introduction), the following locomotory forms could be distinguished: sliding (crawling), swimming, sticking and floating. Sliding meant moving on the glass surface of the container, swimming was defined as moving in the water by slow undulations towards a given direction, whereas floating was determined as a non-moving, immobile state of the animals in the water and sticking was the state of an animal attached motionless for longer time (several minutes) to a surface such as glass wall, stone or a leaf. The first two behaviours were considered as measures of activity, whereas the last two those as passivity.

The specimens of the two experimental groups (*n* = 10 in each) were placed into small (2.5 ml) glass containers containing 10 µM or 100 µM TA diluted in filtered Balaton water. A third group (*n* = 10) served as control and was maintained only in filtered Balaton water. All the animals were exposed to TA and observed individually in every case. An experimental trial lasted for 60 min. Altogether 10 trials were performed at both TA concentrations within a period of a week. Following each 60-min trial, the duration of both the active and passive periods was summarized and their percentage rates were calculated and then averaged.

#### Feeding

To characterize the feeding behaviour of the animals, we applied the already established feeding assay performed on intact *Lymnaea stagnalis* specimens when sucrose solution provided the stimulus evoking consummatory feeding (Kemenes et al. 1986). This behaviour was quantified by its latency (the time duration between the application of feeding stimulus and start of feeding) and the feeding rate (the frequency of the mouth opening/closing cycles). The animals were individually placed into a Petri dish filled with 100 mM sucrose solution dissolved in Balaton water (control) or in a solution containing 10 μM or 100 μM TA in Balaton water (treated animals). When the animals started feeding within 2 min (they responded to sucrose) the feeding latency (in seconds) and feeding frequency (bites/min) were measured. After the feeding assay finished the group of animals (the individual groups separately) were put back in the 15-L container filled with Balaton water until the next day’s testing.

#### Electrophysiological experiments on semi-intact preparations

We also used a simplified, but functional feeding model, a semi-intact preparation consisting of the CNS attached with peripheral nerves to the sensory areas (mouth, lip) of the head region of the animal, meanwhile simultaneously recording the intracellular activity of the buccal feeding neurons (Benjamin and Elliott 1989; Staras et al. 1999a, b). The preparation and recording techniques have been described previously in more detail (Kemenes et al. 1997; Vehovszky et al. 2004). The individual neurons were identified visually by their size, location and intracellular firing pattern after microelectrode penetration (Vehovszky and Elliott 2001). As the feeding stimulus, 100 mM sucrose was dissolved in filtered Balaton water and applied to the lips from a pipette, followed by the application of 10 or 100 μM TA, while the CNS compartment was perfused continuously with normal *Lymnaea* saline (Magoski et al. 1995). Before and after the applications, the lips were washed with filtered Balaton water.

#### Electrophysiological experiments on isolated CNS preparations

For testing the direct cellular effects of TA on feeding neurons, we also recorded their intracellular activity in isolated CNS preparations. The intracellular recording was performed by using filamented (5–8 MΩ resistance) borosilicate glass microelectrodes filled with a mixture of 4 M potassium acetate and 0.4 M potassium chloride. The intracellular signals were recorded by an Axoclamp 2B amplifier, sampled and stored at 1–2 kHz using a National Instruments (NI 6035E) AD converter and a DasyLab 5.6 software.

### Data processing, statistics

To analyse the locomotor activity, the duration spent in active (sliding and swimming) and passive (floating and sticking) periods was measured, then the two (active and passive) categories were summarized separately, and their percentage rate was calculated after each 60-min trial. Thereafter, the results were averaged in (case of) the three experimental groups. Data are presented as mean ± SD.

The frequencies of fictive feeding (F) were calculated by the time intervals in seconds (T) between the rhythmic synaptic inputs recorded from buccal feeding motoneurons (for example N2 inputs on B4 or B8 neurons) as *F* = (1/T)*60; (cycles/min).

Data are presented as mean ± SD. Statistical tests were performed using Student’s *t* test for significance level.

## Results

### Effect of TA on locomotion

**Fig. 1:**
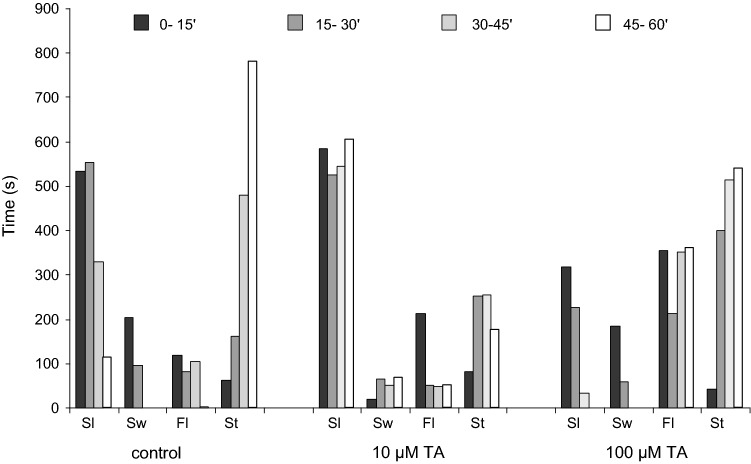
Time and dose-dependent effect of TA on the locomotion of juvenile snails (*Lymnaea stagnalis*). *X* axis shows the different forms of locomotion (*Sl* sliding, *Sw* swimming, *Fl* floating, *St* sticking) in Balaton water (control) and in 10, 100 μM TA. *Y* axis: time spent in a particular form of behaviour during the 900 s (15 min) observation period

**Fig. 2:**
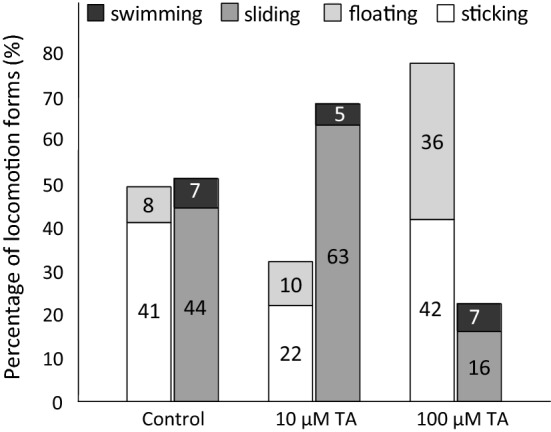
Concentration-dependent effect of TA on the locomotion of juvenile snails (*Lymnaea stagnalis*). In lower concentration (10 μM) TA increased the ratio of active (swimming and sliding) *versus* passive (floating and sticking) periods of locomotion, seen as higher level of sliding activity and with less sticking periods. Higher, 100 μM TA concentration resulted in the predominance of passive forms with almost equal contribution of sticking and floating. Mean ± SD; *n* = 10 in each group. Total time of a trial was 60 min (100%). Numbers indicate the percentage values of each form of locomotion

In the control group, initially sliding prevailed as spontaneous locomotor activity, presumably as a result of the animals monitoring their new environment, but later the number and duration of sticking phases were constantly increasing, reaching nearly the fifty per cent ratio of the whole period recorded (Fig. 1). A relative consistency was observed regarding swimming, in the course of which the snails spent 5–7% of their entire active state in this form of behaviour, independently of the TA concentration applied. The ratio between the time spent in active or passive states was approx. 1:1 (51 vs. 49%) during a 60-min trial (Fig. 2). The animals spent 44% of the trial with sliding and a further 7% with swimming, while they were stuck for 41% and floated 8% of the total time.

Following the application of 10 µM TA, the animals could be separated in two subgroups, according to their displayed activity. The behaviour of the first subgroup (*n* = 5) did not show notable alteration compared to the control, while in the second subgroup (*n* = 5) the snails displayed an elevated level of locomotor activity (hyperactivity), manifested in a more than 40% increase of active periods, compared to the control. The snails spent 92.5% of the time in active phases, within which the sliding activity was 85.5% and the swimming activity made up about 7%. Additionally, the snails remained stuck during 5.5% of the total trial time (60 min), whereas in the rest (2%) they floated. Summarizing the data of the two subgroups (*n* = 10) exposed to 10 µM TA, the active–passive ratio was about 2:1 (68 vs. 32%). The snails spent 63% of the time with sliding, 5% with swimming, 22% with sticking and 10% with floating (Fig. 2).

At high concentration (100 µM) TA treatment, an elevated locomotion dominated the first 15 min of the 60-min observation/exposition period, presumably referring to the escape of the snails from the unpleasant environment. This phenomenon faded away quickly and followed gradually by a general passivity, changing the active/passive ratio strongly for inactivity (Figs. 1, 2). Compared to the 10 µM TA-treated group, a 1:3 (23% vs. 77%) active–passive ratio could be observed. Furthermore, significant changes were also found both in the duration of active and passive states in comparison with the control group (1:3 vs 1:1). The animals were in sliding state for 16% of the total time and spent 7% of the experimental period (60 min) with swimming. The sticking period worked out 42%, whereas this value was 36% concerning floating (Fig. 2).

### Effect of TA on feeding

**Fig. 3:**
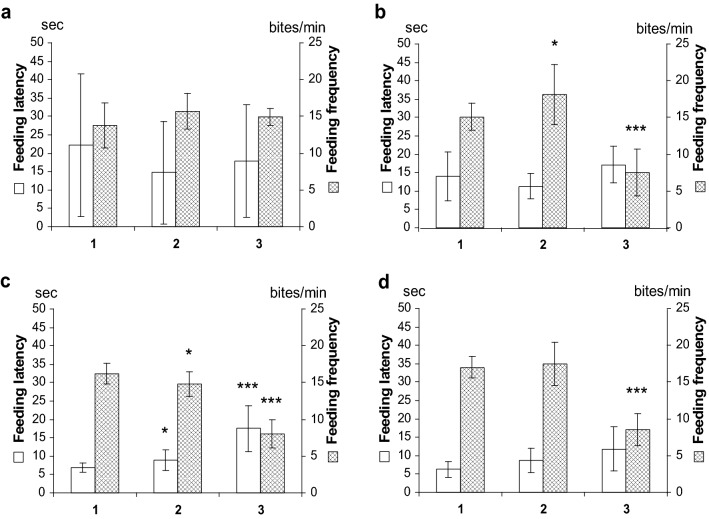
Feeding tests performed on snails following the application of 100 mM sucrose show altered feeding parameters in the presence of TA. **a** All three groups (1, 2, 3) tested in Balaton water show similar, not significantly different feeding latency and feeding rate. **b** An increased feeding rate of group 2 (10 μM TA) and a significantly decreased feeding frequency in group 3 (100 μM TA) are seen compared to the feeding response of control animals tested in water (group 1). **c** Repeated tests next day (1: sucrose; 2: sucrose + 10 μM TA; 3: sucrose + 100 μM TA) show significantly longer latency and lower feeding rate in both treated groups (2, 3). **d** After 48 h all the groups (1, 2, 3) were tested in sucrose only, and group 3 still displayed decreased feeding rate. Left axis shows latency in seconds, and the right axis shows the feeding frequencies expressed as bite/min (mean ± SD; n = 12 in each group). *p < 0.05; **p < 0.01; ***p < 0.001

In preliminary experiments (before testing), three groups of animals (12 specimens in each) were separated and the same groups were tested each consecutive day in sucrose-containing solution, in order to allow them to be accommodated to the experimental conditions. As a result, all the animals responded to sucrose and displayed shorter duration of feeding latency, while the feeding frequencies seemed less scattered (giving smaller error values) during the testing period (Fig. 3a–d).

On the experimental day, the snails were placed in Petri dishes containing either 100 mM sucrose (control, group 1) or mixture of sucrose and freshly dissolved TA (group 2: 10 μM TA, group 3: 100 μM TA, respectively). Although the values of feeding latency were not significantly different in any (1, 2, 3) group (Fig. 3b), members of the 10 μM TA exposed group showed a slightly increased feeding rate (18.13 ± 4.1 bites/min), and the animals tested in 100 μM TA showed a significantly lower feeding frequency (7.5 ± 3.2 bites/min), as compared with the control animals tested on the same day in sucrose (15.1 ± 1.8 bites/min, Fig. 3b, group 1). After the feeding tests, all the animals were placed again in their home tanks filled with Balaton water, and next day the same groups were re-assayed by the same protocol (group 1 in sucrose only, group 2 sucrose in 10 μM TA, and group 3 sucrose in 100 μM TA). All the animals tested in TA showed significantly different feeding parameters from the control group (Fig. 3c). The feeding latency became longer both in 10 μM and 100 μM TA-tested animals (groups 2, 3, 8.9 ± 2. 84 and 17.5 ± 6.3, respectively), compared to the members of the control (group 1, 6.9 ± 1.3 s), and the feeding frequency showed lower values in the TA-treated groups 2 and 3 (14.8 ± 1.67 bites/min and 8.0 ± 6.3 bites/min, respectively), compared to the control group 1 (16.2 ± 1.4 bites/min). All the TA-treated animals were re-assayed again 2 days later in Balaton water containing only sucrose (Fig. 3d). The snails tested in 100 μM TA previously (group 3) still showed significantly lower feeding rate compared to the controls (8.58 ± 2.2 bites/min and 17.0 ± 1.47 bites/min, respectively).

### Effect of TA on semi-intact preparations

**Fig. 4:**
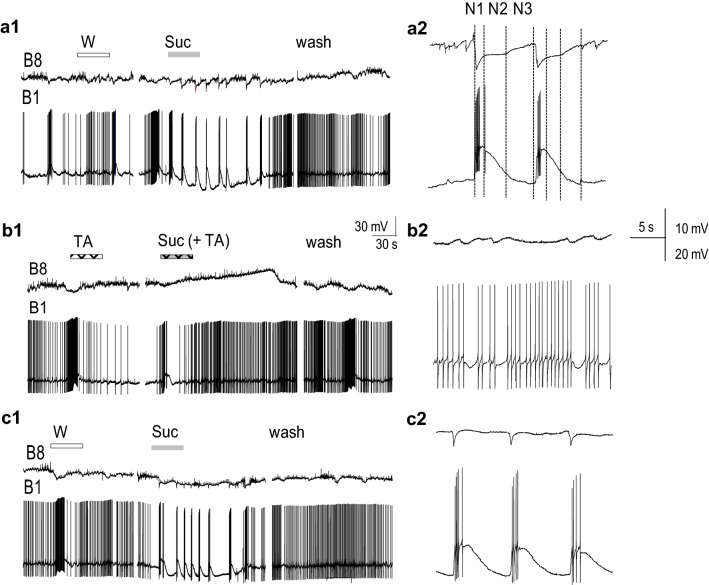
Preliminary application of TA prevents sucrose response. A Simultaneous recording from two buccal motoneurons (B8, B1) during Balaton water application (W), followed by 100 mM sucrose (Suc) applied to the lip area (**a1**). Sucrose-evoked rhythmic activity is seen as series of inhibitory inputs on B8 neuron and depolarization with action potentials on B1 neuron, respectively. N1, N2, N3 indicate the phases of fictive feeding (**a2**). B Application of 100 μM TA did not change the spontaneous activity, and sucrose dissolved in TA solution (Suc +TA) fails to evoke the feeding rhythm (**b1**), as rhythmic synaptic inputs are not visible in B8 or B1 neurons (**b2**). C After 20-min washing, the lip with water the sucrose-evoked intracellular response recovered (**c1**) and the cyclic synaptic inputs re-appear in the intracellular activities of feeding neurons. (**c2**). Representative example of the experiments carried out on 6 independent preparations

On semi-intact (head-CNS) preparations, first we tested the peripheral sensory-central pathways by applying 100 mM sucrose to the chemosensory head areas (lip, mouth). A positive intracellular feeding response was considered when the identified feeding neurons displayed the rhythmic pattern of fictive feeding (sequence of synaptic inputs or firing activity) immediately after sucrose application as seen in Fig. 4, which represent an example of the 6 experiments out of 8 preparations, when sucrose response was evoked successfully and repeatedly.

Without peripheral stimulation, the buccal feeding neurons were characterized by a rather irregular activity pattern, and water applied to the lip (representing a tactile stimulus) did not alter their intracellular activity (Fig. 4a1, c1). After application of 100 mM sucrose solution onto the lip area, the same neurons displayed a synchronous, rhythmic activity formed by a series of (excitatory and inhibitory) synaptic inputs, indicating activation of the CPG feeding network (Fig. 4a1). For example regular inhibitory (N1, N2 phase) inputs were recorded from the B8 motoneuron, simultaneously with the strong (N1 phase) excitatory inputs (depolarization and bursting) on the B1 motoneurons (Fig. 4a2).

Applying 100 μM TA onto the lip-mouth area did not alter the spontaneous intracellular pattern of feeding neurons, but prevented the intracellular feeding response of the neurons after sucrose application (Fig. 4b1, 2). As a result, the activity pattern of individual feeding neurons did not display inputs of fictive feeding, indicating that sucrose failed to trigger the neuronal network of the feeding CPG system (Fig. 4b2). The feeding response of the central neurons only partially recovered after the lip–mouth area was washed by Balaton water for 20 min before the application of sucrose was repeated again (Fig. 4c1, 2).

### Effect of TA on isolated CNS preparation

Although both the feeding tests carried out on intact animals, and the electrophysiological results obtained from semi-intact preparations suggest modulation of the sensory pathways by TA, it could not be excluded that TA also directly targets the interneurons of the central feeding CGP network, resulting in their decreased ability to evoke feeding pattern.

**Fig. 5:**
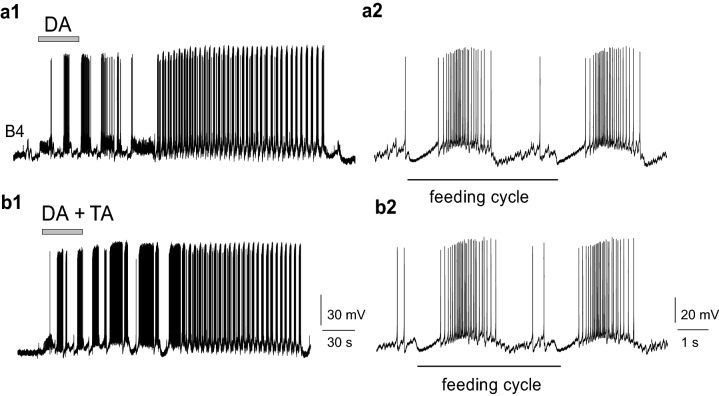
In isolated central nervous system TA did not prevent fictive feeding evoked by 1 mM DA application. **a1** The feeding pattern recorded on the B4 buccal motoneuron in saline; **b1** intracellular activity in the presence of 100 μM TA. The feeding cycles marked by the intervals between the N2 phase synaptic inputs on B4 motoneuron (**a2**) does not show significant changes after DA application (**b2**). Representative example of independent experiments carried out on 6 preparations

To activate the central feeding (CPG) network in the isolated CNS preparation, we performed a drop application of 100 μl of 1 mM DA directly in the experimental chamber during continuous perfusion with normal saline (Fig. 5, a representative example of independent experiments on 6 preparations). This kind of application enabled a phasic concentration change of DA and washing out of the transmitter effect in 4–5 min. Activation of the CPG network by DA application was well detected by the altered activity of feeding motoneurons as rhythmic pattern of synaptic inputs and bursts of action potentials appeared (Fig. 5a1). This dopamine-evoked rhythmic activity, for example a series of N2 phase synaptic inputs on B4 motoneuron (Fig. 5a2), formed a similar pattern as we recorded in semi-intact preparations when sucrose was applied to the lip area (Fig. 4a2). TA (100 μM) applied in the perfusion chamber did not change the spontaneous activity or the DA evoked cellular responses of the feeding neurons. The frequency values of the spontaneous activity did not show significant differences in normal saline, in 100 μM TA or after washing out with saline again (4.0 ± 1.0, 5.0 ± 0.7 and 4.1 ± 0.9 cycles/min, respectively). Neither was the dopamine-evoked fictive feeding modulated by the presence of TA in the perfusion chamber, as the frequency of feeding rhythm (measured by the length of the feeding cycles) did not change either (Fig. 5b1, b2).

**Fig. 6:**
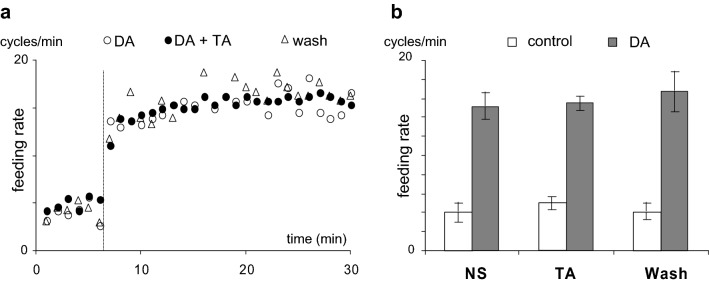
**a** In isolated central nervous system TA did not alter the frequencies of DA evoked fictive feeding. **a** Prior to DA application (control) the spontaneous rhythm of intracellular activity (with frequencies around 5 cycles/min) is switched to a threefold frequency increase after applying 100 μl 1 mM DA in the perfusion chamber (dotted line). **b** Diagrams showing no significant differences in frequencies in normal saline (NS), in the presence of 100 μM TA, or after washing with saline again (Wash)

While comparing the top frequencies of DA-evoked fictive feeding, no significant differences were seen in normal saline (control), in the presence of 100 μM TA or after washing out (15.0 ± 1.29; 15.48 ± 1.18; and 16.7 ± 2.1 cycles/min, respectively (Fig. 6a, b).

## Discussion

### Behavioural alterations evoked by TA on snails

Our experimental results revealed dose-dependent (both stimulatory and inhibitory) effects of TA on the behaviour (locomotion and feeding) of the pond snail, *Lymnaea stagnalis*, following the application of this allelochemical. The lower (10 µM) concentration of TA applied probably is about the individual (stimulus-) threshold concentration, as half of the animals did not show differences in their locomotory activity compared with the control group, while in the other group of the animals the spontaneous locomotion was slightly stimulated. Additionally, the frequency of the sucrose-evoked feeding response (feeding rate) was also significantly increased in 10 µM TA. A weakly facilitated locomotory activity of snails was also observed initially, when high (100 µM) concentration of TA was applied, particularly during the first 15-min period of exposure, but later TA inhibited the locomotion. Higher concentration (100 µM) TA also reduced the feeding, resulting in longer feeding latency and lower biting frequency values of intact animals. Consequently, high concentration (100 µM) of TA acted as an inhibitor of both locomotion and feeding, resulting in a long-lasting non-moving, passive state of the exposed animals, and this concentration of TA also decreased feeding activity of the intact animals.

The neuromodulator role of monoamines has well been established in the regulation of snail locomotion (see Pavlova 2019). Sliding (crawling), which is characteristic for *Lymnaea*, is based on the activity of the ciliated epithelial cells of the sole, and ciliary movement is modulated by serotonergic mechanisms originating from the central (pedal) neurons (Sidorov and Kazakevich 2001; Pavlova 2010). The facilitatory role of serotonin has also been demonstrated during the late period of *Lymnaea* embryogenesis (Filla et al. 2009).

It is assumed that TA acts directly on the ciliary activity either by directly influencing the 5-HT receptors or hitting the serotonergic regulatory pathway(s) through an afferent-efferent system, including receptor cells located on the surface of the foot and the central (pedal) serotonergic efferent elements. The slow forwarding swimming movement of the juvenile snails, observed in our present study, may also be influenced by TA directly on the foot musculature. In *Lymnaea* contractions of the foot musculature are primarily controlled by dopaminergic elements of the nervous system (Pavlova 2013, 2019), and the involvement of acetylcholine (ACh) in the snail muscular activity was also suggested, particularly, in terrestrial snails (Walker and Holden-Dye 1991; Panchin et al. 1995; Krajcs et al. 2014). Most recently TA was shown to inhibit acetylcholinesterase (AChE) enzyme activity in vitro (Kim et al. 2018); therefore, we may assume that TA also affects the cholinergic neuromuscular transmission in *Lymnaea* as well. Activity of AChE eliminates the released neurotransmitter from the synaptic cleft; therefore, decreased AChE activity may initially result in enhanced ACh-mediated stimulatory effects, followed by the impairment all the neuronal mechanisms where cholinergic neurotransmission is involved, acting both centrally and on the peripheral muscles. We cannot exclude, therefore, that the compound behavioural alterations we observed in the presence of TA (an initial stimulation followed by decreased activity) were the results of the above-mentioned biphasic effect of AChE inhibition.

The feeding assay we applied (Kemenes et al. 1986) allows the analysis of both the appetitive and consummatory phases of feeding controlled by peripheral and central neuronal mechanisms. Feeding latency is primarily determined by activation of the chemoreceptors around the lip and the chemosensory pathways projecting to the CNS. The rhythmically organized feeding movements (rasping) during the consummatory phase are executed by the buccal musculature, which cyclic activity is driven by the CPG network in the CNS (Benjamin 1983). The two components of feeding can be selectively affected by specific neurotoxins. For example, treatment of intact animals by rotenone, a dopaminergic neurotoxin, did not alter the feeding latency but decreased the feeding rate suggesting its target is likely located centrally, as part of the dopaminergic system in the CNS (Vehovszky et al. 2007). Therefore, our feeding results on intact animals showing that both latency and feeding rate were altered in the presence of TA initially suggested inhibitory effects of both the peripheral (sensory) and central mechanisms regulating the feeding behaviour of *Lymnaea*.

### Neurophysiological changes in the feeding system evoked by TA

Our experiments on semi-intact preparations demonstrated that application of TA prior to the sucrose feeding test inhibited the appearance of the neuronal feeding response (rhythmic pattern of intracellular activity) to sucrose. This lack of sucrose response shows that the CPG network did not trigger the feeding rhythm, either due to peripheral impairment (receptor blocking) or direct central inhibitory mechanisms. TA has a bitter taste similarly to quinine and, therefore, may represent a negative feeding stimulus for *Lymnaea* (Staras et al. 1999a, b). Supporting the second option (inhibition of central processes by TA effect), a very recent work suggests the presence of subpopulations of chemosensory neurons, which may transmit direct (both stimulatory and inhibitory) sensory modalities to the CPG network in *Lymnaea* (Straub et al. 2004).

Electrophysiological results, moreover, also revealed that TA did not alter the potential of the pattern generating interneurons to trigger feeding activity in the central network. Dopamine applied on the isolated CNS evoked fictive feeding in the presence of 100 µM TA, and this rhythmic activity showed the same qualitative and quantitative characteristics as recorded in normal saline. Consequently, we can exclude a direct effect of TA on the central network of feeding activity, responsible for the weaker neuronal response to sucrose in semi-intact preparations or lower feeding rate of intact animals. Instead, it is more likely, that TA blocks the peripheral afferent pathways running to the CNS, by inhibiting the chemosensory receptors involved in feeding. This is also suggested by literature data obtained on insect larvae which demonstrated the blocking effect of tannins on peripheral sucrose sensitive sensory cells (Denno and McClure 1983; Dethier 1982; Panzuto et al. 2002). Additionally, as we already mentioned for locomotion above, we may also assume a direct (AChE blocking) effect of TA on the buccal musculature, which cholinergic innervation has also been described in different snails (Yoshida and Kobayashi 1991; Jordan et al. 1993).

In summary, we conclude that allelochemicals of plant origin may be an important, yet little investigated, component affecting the behaviour of the invertebrate fauna living in the aquatic environment, hence influencing the complex life of the entire ecosystem in the long term. Accordingly, our future research would include double aims. First, the putative effect(s) of tannins extracted from endemic plant species of Lake Balaton on different behaviours (feeding, locomotion) of *Lymnaea stagnalis* will be studied. Second, we see a potential to widen our analysis on other, non-gastropod invertebrate, first of all zooplankton species, as well as the ruling biofouling invasive bivalve, *Dreissena* sp.
